# 

**Published:** 1916-06

**Authors:** 


### Publishers' Announcement

The furnishing of a modern dental office is a subject of which the manufacturers of dental equipment have made a special study and the present offering of items necessary for an office are superb. The dental chair has assumed a graceful outline that shows the hand of art enclosing the already perfected mechanical parts; the dental cabinet shows also the touch of artistic design together with a skillful allotment of all available space for the various instruments and materials it is necessary for it to hold; the electrical equipment now so necessary and essential to an office is perfectly made and has the precision of smooth-running and efficient service as well as important appearance. The patient is much interested in the apparatus of a dental office and is favorably impressed by the modern equipment.

The DENTAL REGISTER is much interested in the modern equipment of the modern dental office and has within its covers the leading manufacturers' advertisements which will help you select good reliable equipment of latest design.

### ANNOUNCEMENTS

NATIONAL DENTAL ASSOCIATION. The annual meeting will be held in Louisville, July 25 to 28. The program is published elsewhere in this issue.

---

AMERICAN SOCIETY OF ORTHODONTISTS will hold its annual meeting in Pittsburg, July 20 to 22, 1916.

---

NATIONAL ASSOCIATION OF DENTAL EXAMINERS will hold its annual session in Louisville, July 28 to 29, 1916.

---

NATIONAL ASSOCIATION OF DENTAL FACULTIES will hold its next meeting in Louisville, July 22 to 24, 1916.

---

WEST VIRGINIA EXAMINERS. The next meeting of the West Virginia State Board of Dental Examiners for examination of applicants, will be held in Wheeling, W. Va., on June 27, 28 and 29, 1916.

For blanks and further information, address Dr. H. H. Smallridge, Secretary, Charleston, W. Va.

---

LOUISIANA DENTAL SOCIETY. At the thirty-eighth annual meeting of the Louisiana State Dental Society, held in Lake Charles, the following officers were elected for the ensuing year:

Paul N. Cyr, President; A. K. Fort, First Vice-President; C. S. Tuller, Second Vice-President; J. Crimen Zeidler, Secretary; J. A. Gorman, Corresponding Secretary; O. J. Ory, Treasurer.

New Orleans was selected as the next meeting place and in view of the fact that a meeting is being planned comprising the dental societies of Alabama, Louisiana, Mississippi and Texas, this gives promise of being one of the largest meetings ever held in this part of the country in quite a number of years.

